# The decreasing availability of reindeer forage in boreal forests during snow cover periods: A Sámi pastoral landscape perspective in Sweden

**DOI:** 10.1007/s13280-022-01752-w

**Published:** 2022-06-21

**Authors:** David Harnesk

**Affiliations:** Lund University Centre for Sustainability Studies (LUCSUS), PO Box 170, 22 100 Lund, Sweden

**Keywords:** Equilibrium theory, Forestry, Lichens, Non-equilibrium theory, Reindeer husbandry, Sámi pastoralism

## Abstract

This paper argues that Sámi reindeer pastoralism in Sweden is highly stressed during the critical snow cover periods due to large-scale human interventions, especially forestry, and that these have over time significantly worsened the ecological conditions for natural grazing-based responses to changing snow conditions caused by climate change. Informed by a literature review, the paper conceptualises two, overlapping ecological dynamics that shape the availability of lichen as key forage resources within a Sámi pastoral landscape perspective: the grazing dynamics of reindeer during snow cover periods as determined by climatic stochasticity, and the more predictable vegetation dynamics of lichen habitat formation, growth and sustenance based on structured forestry practices. This could help articulate an intervention ecology that pursues sustainable ecological conditions for natural grazing-based Sámi reindeer pastoralism, along with other goals. As such alternatives are likely to face political resistance, the article discusses the implications of its findings within a science–politics interface.

**Supplementary Information:**

The online version contains supplementary material available at 10.1007/s13280-022-01752-w.

## Introduction

Boreal forests in Sweden have been subject to large-scale human intervention and climate change, and as a result, their biophysical features have been significantly altered by societal forces (Östlund et al. 1997; Gauthier et al. 2015; Svensson et al. 2019). Although many land uses have contributed to this transformation, intensive forestry has been a major force as it is practiced across 90% of the forest cover (Gauthier et al. 2015). While land uses can be interpreted and evaluated from different theoretical perspectives and normative positions, the present article focuses on how they, with impacts at multiple ecological scales, decrease the availability of lichens as key forage resources for Sámi reindeer pastoralism during snow cover periods—“a critical bottle neck in the annual herding cycle, as they are crucial to reindeer survival and calving success” (Axelsson-Linkowski et al. 2020: p. 482).

Informed by a literature review, the aim of this paper is to conceptualise the main ecological dynamics that have reduced the availability of lichens for reindeer during snow cover periods. Within a Sámi pastoral landscape perspective (Horstkotte et al. 2014; Benjaminsen et al. 2015), I argue that there are two, overlapping ecological dynamics that shape the forage availability problem. The first concerns the grazing dynamics of reindeer during snow cover periods as determined by climatic stochasticity, conceptualised with non-equilibrium theory. The second relates to the more predictable vegetation dynamics of lichen habitat formation, growth and sustenance based on structured forestry practices, conceptualised with equilibrium theory. Finally, I discuss how such ecological reasoning could inform an intervention ecology, with theoretically informed goal-driven interventions at different (social–)ecological scales (Hobbs et al. 2011). Such interventions would support a multi-purpose forest landscape conducive to both natural grazing-based Sámi reindeer pastoralism and other goals that people could mobilize to achieve.

My contribution is made within the academic literature that tends to study Sámi reindeer pastoralism in Sweden with equilibrium theory (e.g. Uboni et al. 2020), or indirectly incorporate non-equilibrium dynamics via the experiences of Sámi pastoralists (e.g. Axelsson-Linkowski et al. 2020; Rosqvist et al. 2021), or examines it broadly through the concept of resilience (e.g. Moen and Keskitalo 2010). I also contribute to discussions on how ecological research, while making scientific contributions, has political implications when it informs land use practice and planning (see Jacobs et al. 2018)—notably in a context where deterministic paradigms uncritically promote controlling stochasticity by labour and capital inputs (such as work, feed and fuel) despite that such measures may express emerging critical states for natural grazing-based pastoralism. It also helps clarify how methodological decisions in (social–)ecological research connect to political tensions that are rooted in how material interests and social relations interact with the bio–geo-physical world (Harnesk and Isgren 2021; Longo et al. 2021)—which is relevant to wider discussions on sustainable land use planning.

I start by presenting my methodology. Next, I outline a Sámi pastoral landscape perspective in Sweden, using Sámi terminology in the Northern Sámi language. Then, I present a dialogue between equilibrium and non-equilibrium theories, focusing on ground and pendulous lichens located in boreal forests as key forage resources for reindeer during snow cover periods. I argue that equilibrium theory better captures vegetation dynamics of lichens in relation to forestry, and that non-equilibrium theory better captures grazing dynamics of reindeer in relation to Sámi reindeer pastoralism. From this, I argue these conceptualisations can be combined to articulate a multi-purpose forest intervention ecology. I conclude by discussing how such alternative sustainable land use planning approaches could help mobilize broader political support at the interface between science and politics.

## Methodology

My literature review was informed by the method of immanent criticism (Isaksen 2018). The purpose of an immanent critique is to depart from an object of study (e.g. theories or practices) and identify any internal gaps and limitations (e.g. theory–theory, theory–practice and/or theory–data inconsistencies) in order to articulate more comprehensive explanations and better practices (*ibid.*). The method establishes a process for identifying the limits of existing explanations and practices, and for understanding when, where and how to look beyond them (*ibid.*).

Immanent critique is particularly useful when there are two competing perspectives that relate to a concrete phenomenon (Isaksen 2018). In the present study, the concrete phenomenon is the problem of decreasing availability of reindeer forage during snow cover periods in Sweden. I draw upon both equilibrium and non-equilibrium theories, found in the rangeland ecology debate, where Briske et al. (2003) suggest asking when equilibrium and non-equilibrium dynamics apply, and at what scales, rather than expecting one paradigm to be able to comprehensibly explain rangelands. These two paradigms provide the competing perspectives required for immanent critique, here focusing on the ecosystem and landscape dynamics that impact the amount and accessibility of ground and pendulous lichens for reindeer during snow cover periods.

Based on their relevance to the problem definition and the two competing perspectives, I reviewed academic literature on lichens and Sámi reindeer pastoralism in Sweden. This provided an overview of the current state of academic knowledge regarding the factors that affect the availability of lichens as reindeer forage during snow cover periods. The search examined two databases, Web of Science and Scopus, for the time period 1 January 1990 to 28 February 2022, using the terms: (1) “lichen” OR “snow” OR “ice”, (2) “Sweden” or “Swedish”, (3) “reindeer husbandry” OR “reindeer herding” OR “reindeer pastoralism” OR “forestry” OR “forest management”. A review of 542 abstracts resulted in 34 articles being reviewed in full, while a further 54 sources (articles, statistics and GIS data) were added from a review of citations in the selected articles (see Supplementary Material).

## A Sámi pastoral landscape perspective

Pastoralism is a land use based on livestock that makes use of what are often considered marginal environments that are unsuitable for agricultural production in productive and sustainable ways if flexible mobility can be maintained (Scoones 2021). Pastoralists are experts at embracing turbulence, uncertainty and complexity as throughout history they have responded to highly variable environments, market volatility and uncertain institutional and political conditions (*ibid*.). Their adaptive responses to stochastic events are sophisticated, and often based on traditional and experience-based knowledge systems and mutual and community support systems (*ibid.*).

**Fig. 1:**
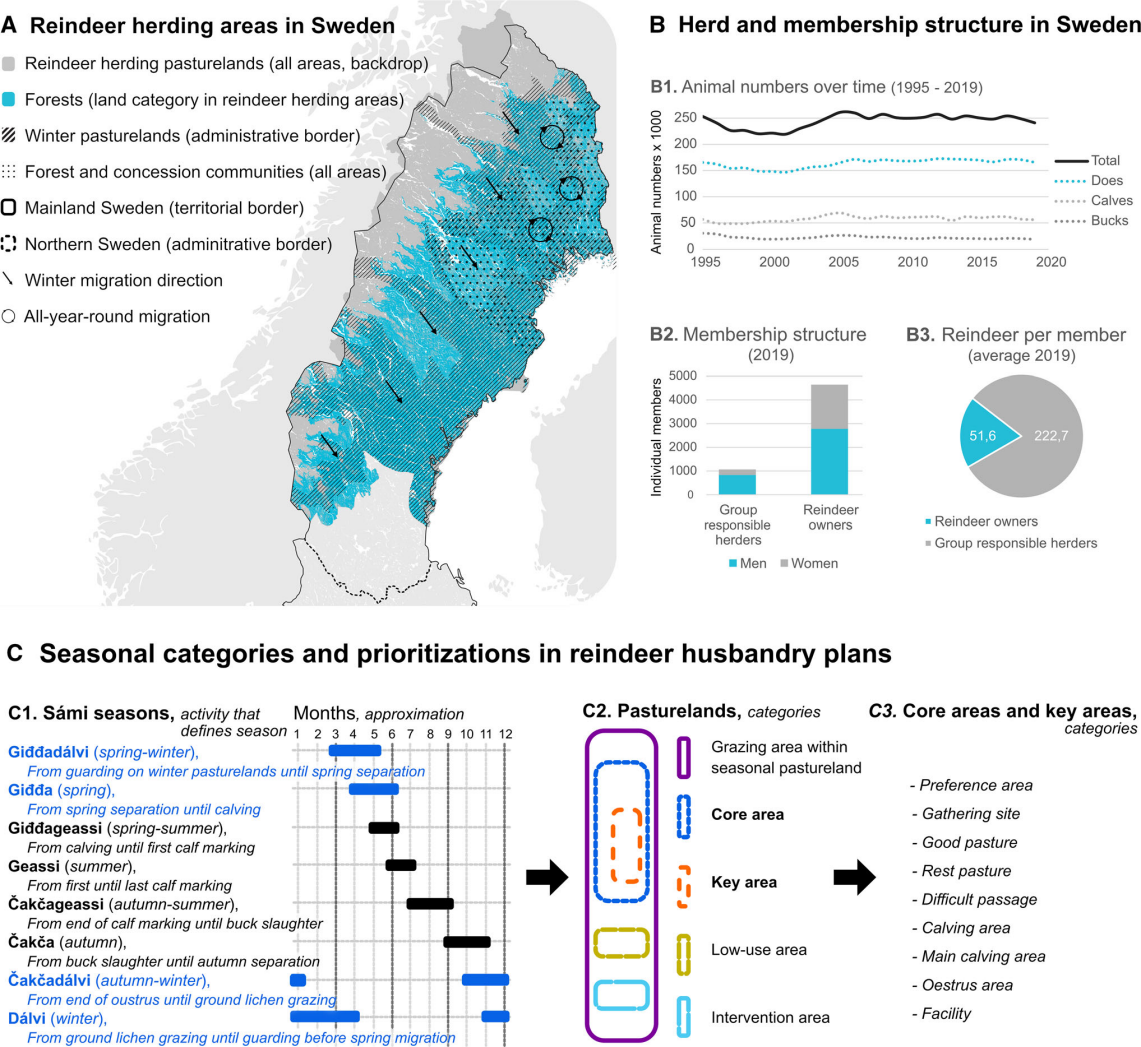
Overview of Sámi reindeer pastoralism in Sweden. **A**: Map of reindeer herding areas in Sweden based on GIS data used in reindeer husbandry plans (Jougda 2017). **B**: Herd and membership structure of reindeer herding communities (Sámediggi 2020). **C**: Categories for seasons, pasturelands, core areas and key areas within reindeer husbandry plans (Jougda 2017). Blue text signifies snow cover periods

In Sweden, Sámi reindeer pastoralism is a livelihood and cultural practice based on natural grazing semi-domesticated reindeer, and is practiced by and within ‘reindeer herding communities’ (RHCs; Sámediggi 2021a). The latter are formal organisations that consist of reindeer owners and (group responsible) herders who practice pastoralism within their corresponding geographical area, which sometimes overlaps with neighbouring RHCs (*ibid.*; see Fig. 1A, B). However, RHCs are more accurately captured in more holistic and non-economic community conceptualisations (e.g. Buchanan et al. 2016), and are also of wider cultural importance to the indigenous Sámi people (Sámediggi 2021a).

Sámi reindeer pastoralism is practiced within pastoral landscapes (Benjaminsen et al. 2015) where land uses are organised after historical seasonal migration patterns, and specific areas represent different and multiple functions for each of the eight Sámi pastoral seasons (Sandström et al. 2003; Horstkotte et al. 2014; Jougda 2017; see Fig. 1C for definitions of the seasons). This form of pastoralism requires multi-purpose landscapes that provide the functions necessary for reindeer survival and reproduction, including the ability to adapt and migrate depending on weather conditions (Horstkotte et al. 2014, 2017). This paper focuses on a narrow yet important set of ecological dynamics that determine forage availability during snow cover periods on related seasonal pasturelands (see Fig. 1C).

Pasturelands have been categorised with respect to their functions by RHCs in participatory GIS planning documents called ‘reindeer husbandry plans’ (Jougda 2017). These plans include categories such as the Sámi pastoral seasons, the relative degree of importance of different pasturelands, and areas that need to be improved through interventions (*ibid.*; see Fig. 1C). A review of these documents highlights that landscape functions are highly interconnected, not only with one another, but also with other functions beyond the scope of this study (e.g. migration pathways, resting areas, calving and oestrus areas). It is therefore necessary to consider the cumulative effects of changes across the Sámi pastoral landscape when examining the ecological conditions for reindeer pastoralism (e.g. Kløcker-Larsen et al. 2016).

Sámi reindeer pastoralism is planned and managed by RHCs and groups within them, called *siida* (Sámediggi 2021a). Grazing patterns in pastoral landscapes are determined by reindeer and pastoralists together within local (patch/feeding site, hours/minutes) and intermediate (feeding areas, days/weeks/months) scales, within and across each of the eight seasonal pasturelands (Skarin and Åhman 2014; Axelsson-Linkowski et al. 2020). The factors that shape these practices are incredibly dynamic, captured in the Sámi concept of *jahkodat*, which highlights “the distinctiveness of any given year, not as a mutually interchangeable unit of time, but as a particular and unique succession of specific conditions, with variable and cumulative effects” (Benjaminsen et al. 2015, p. 226; see also Horstkotte et al. 2017).

Different types of RHCs exist and they have different land use strategies for grazing (Sámediggi 2021a). In ‘mountain RHCs’, summer pastures are characterised by grasses and herbs in mountainous areas, autumn pastures may also involve mushrooms in the mountainous forests, while the arrival of snow during late-autumn eventually shifts the reindeer’s diet to mainly consist of lichens in forests that are located further to the east(–south-east) (*ibid.*). ‘Forest RHCs’ and ‘concession RHCs’ generally remain in forests throughout the year (*ibid.*). The former also migrate east(–south-east) for winter, while the latter remains confined to a more limited area with shorter migrations (*ibid.*). The Sámi parliament considers the following conditions to be conducive to natural grazing: pasture availability (i.e. amount/accessibility), grazing peace (i.e. the ability to graze without threats/disturbances) and climatic conditions (e.g. seasonal temperature/precipitation patterns) (*ibid.*).

Sámi reindeer pastoralism has been practiced for centuries across Sápmi, which refers to the territory of the indigenous Sámi people, and that spans across Norway, Sweden, Finland and Russia (Hansen and Olsen 2014). The current form found in Sweden emerged in the mid-1900s (an era of profound social and environmental change), when the pre-existing intensive form of mainly subsistence production shifted to an extensive form of both subsistence and commercial production (Beach 1990). Sámi pastoralists began to spend less time in close proximity to their reindeer, and instead had them range more freely (*ibid.*). Over time, herd structures were changed, and new land use strategies, enabled by mechanisation, were adopted (Riseth et al. 2016; Uboni et al. 2020). Nowadays, Sámi pastoralists use snowmobiles, all-terrain vehicles, helicopters and trucks, and many reindeer are equipped with GPS collars (*ibid.*). This change has been partly driven by the Swedish state’s ‘rationalisation programme’, which was informed by management ideas on commercial livestock husbandry (Beach 1990), found in the rangeland ecology debate in North America (Sayre 2017). However, it has not necessarily been fully adopted, and certainly not without resistance (Beach 1981).

## The balance of nature: Equilibrium theory and vegetation dynamics

Equilibrium theory is captured in the metaphor of the balance in nature. The equilibrium paradigm assumes that abiotic patterns are relatively constant; plant–herbivore interactions are tightly coupled and subject to biotic regulation; population patterns follow carrying capacity and are density dependent, and community/ecosystem characteristics are structured by competition and internally regulated (Ellis and Swift 1988; Briske et al. 2003).

**Fig. 2:**
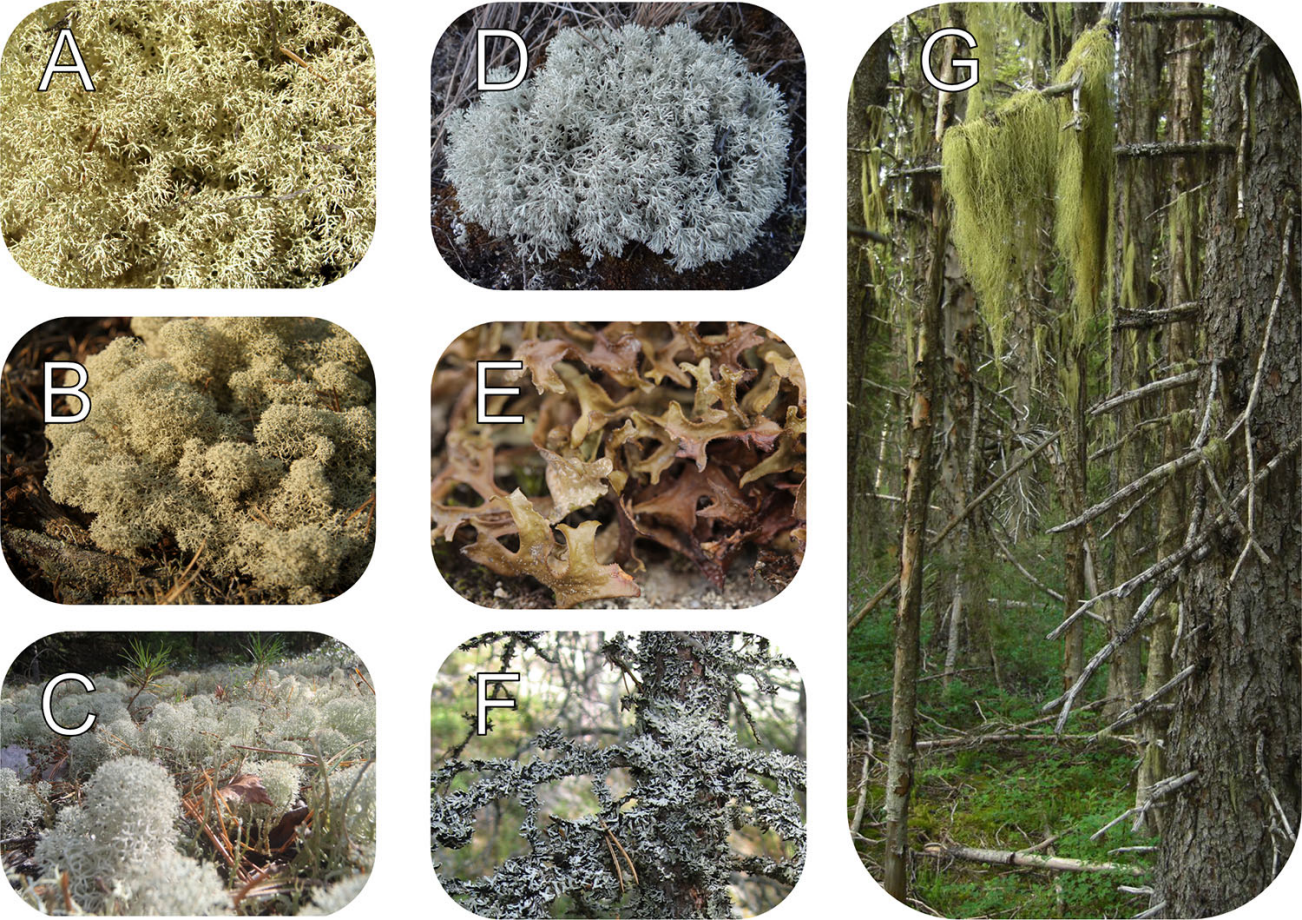
Lichens commonly foraged by reindeer during snow cover periods. **A**: *Cladina arbuscula* (*jeagil*). **B**: *Cladina rangiferina* (*jeagil*). **C**: *Cladina stellaris* (*jeagil*). **D**: *Cladina mitis* (*jeagil*). **E**: *Cetraria islandica* (*jeagil*). **F**: *Hypogymnia physodes* (*gatna*). **G**: *Bryoria fuscescens* (dark green *lahppu*) and *Alectoria sarmentosa* (bright green *lahppu*). Photos: Wikimedia Commons

Lichens are the focus of my study, as they are the key forage resource for reindeer, especially between *dálvi* and *giđđa* (Heggberget et al. 2002; see Fig. 1C for definitions of the seasons), although other vegetation types also contribute to their diet during that period (Åhman and White 2018). Reindeer predominately feed on ground lichens, *jeagil*, pendulous (alectorioid) lichens epiphytic on trees, *lahppu*, and, to a lesser extent (and hence omitted from the article), foliose lichens epiphytic on trees on trees and crustose lichens on rocks, *gatna* (Inga 2009; Fig. 2). Habitat formation, and the growth and sustenance of these lichens have been studied extensively with successional theory (e.g. Esseen et al. 1997; Dettki and Esseen 1998; Horstkotte and Moen 2019), located within the equilibrium paradigm (Sayre 2017). Consequently, we know a lot about them, and how different forestry practices affect their amount and distribution in boreal forests.

### Lichens and successional theory

Lichens are symbiotic organisms that consist of a fungal heterotrophic partner and algae or cyanobacteria autotrophic partner(s) (Nash 2008). The fungal partner provides structure and absorbs nutrition from water sources, while the algae/cyanobacterial partner produces carbohydrates through photosynthetic activity (*ibid.*). Lichens have no organs that allow for direct nutrient uptake from soils, but have outgrowths that allow them to attach to surfaces (*ibid.*). They have poor dispersal ability and relatively low growth rates, but thrive where other organisms struggle to survive (*ibid.*).

Ground lichens gain biomass slowly as growth is restricted to times when they are wet, and their dispersal ability is poor, as they mainly depend on fragmentation (Nash 2008). Lichens require light, a suitable combination of water and temperature and a balance of nutrients to grow effectively (Gaio-Oliveira et al. 2006). At the level of bottom-layer vegetation, they mainly compete against mosses and vascular plants, and have a competitive advantage on dry soils as they source nutrients from moisture and elements in the air (Horstkotte and Moen 2019). At local scales of forest structure, dense canopies create light-limiting conditions, and therefore, open-canopy forests better support lichen recovery and dominance (Jonsson Čabrajič et al. 2010; Jonsson et al. 2021). Therefore, so-called pine-heaths (coniferous forests on dry soil dominated by Scots pine) tend to be most conducive to ground lichen dominance (see Horstkotte and Moen 2019).

Pendulous lichens have similar growth requirements and poor dispersal abilities (Dettki et al. 2000), but also depend on specific substrates to survive (Esseen et al. 1996). At the tree scale, factors such as tree age, branch size and nutrient availability determine growth, while at the stand scale, important factors include stand age, micro-climate and the vertical position of lichens within the canopy (Coxson and Coyle 2003; Sillett and Antoine 2004). Important features in the spatial structure of the landscape that influence colonisation, growth and mortality include forest patch size, distance to forest edge and edge orientation (Esseen 2019). The large surface area to mass ratios of lichens, together with how they filter moisture and elements from the air, make them sensitive to air pollution, and climate change may shift their broad-scale distribution (Esseen et al. 2016). Taken together, community formation depends on forest age and possibly even forest volume (Dettki and Esseen 2003; Jaakkola et al. 2006). These conditions are most commonly found in continuous old-growth (over 100 years) forests (Esseen et al. 1996; Boudreault et al. 2009), although limited amounts can be found in stands older than 60 years (Horstkotte et al. 2011).

Disturbances that cause biomass removal/destruction (e.g. forest fires and forestry) alter habitat structure and functions, with different impacts on lichen communities. At the ground vegetation level, mosses and vascular plants can outcompete ground lichens during early stages of succession; on the other hand, when dense forest stands dominated by competitors are subject to disturbances there can be opportunities for ground lichens to colonise (Horstkotte and Moen 2019). It is possible that, prior to the 1900s, Sámi pastoralists used forest fires to remove mosses and vascular plants to promote lichens (Hörnberg et al. 2018; Cogos et al. 2019). As pendulous lichens require trees to exist, they disappear after disturbances, but established communities can colonise neighbouring stands that have recently been subject to disturbance (Dettki et al. 2000), limited by distance from the edge of mature trees (Stevenson 1990).

### Lichens over time and space

Ground lichen abundance and distribution over time has been examined at the macro level with data from the Swedish National Forestry Inventory at sample plots within RHCs (Sandström et al. 2016; Horstkotte and Moen 2019). Sandström et al. (2016) concluded that lichen ground cover in productive forest land had significantly decreased between the 1950s and the 2010s. Notably, there was a significant reduction of plots with over 50% lichen ground cover (a fall from 13 to 3.7%) (*ibid.*). Hortskotte and Moen (2019) concluded a loss of plots with over 25% lichen ground cover between the 1980s and the 2010s (a fall from 70 to 45%). Studies at spatial scales relevant to individual RHCs report similar loss trajectories (see Berg et al. 2008). The data used in these studies, however, do not include lichen height, which could provide better estimates of lichen biomass (Uboni et al. 2019).

Pendulous lichens are difficult to collect data on and studies use proxies or models to estimate changes in abundance and distribution. In particular, stand age is often used as a proxy to assess pendulous lichen occurrence (Boudreault et al. 2009). Between 1955 and 2017, in productive forest land across northern Sweden, there have been falls in both forests older than 60 years (from 59 to 36%), and forests older than 100 years (from 28 to 19%) (Swedish National Forestry Inventory 2021a). At the landscape scale, Horstkotte et al. (2011) concluded that forest cover older than 60 years fell from 84 to 34% between 1920 and 2006. Berg et al. (2008) looked at the same landscape, and estimated a decrease in mean stand age from over 200 to 70 years throughout the 1900s. Dettki and Esseen (2003) combined lichen litter sampling and models based on historical forest data; their study identified a reduction in total lichen biomass between 1959 and 1999 (from 12 to 4.6 g/m^2^) (see also Dettki and Esseen 1998). Areas with high amounts of anthropogenic airborne sulphur and nitrogen emissions may also be associated with lower amounts of pendulous lichens (Esseen et al. 2016).

The absolute losses described above are coupled with increased fragmentation of lichen habitats (Kivinen et al. 2010, 2012; Svensson et al. 2019). The regional-scale study reported in Svensson et al. (2019) identified significant losses of intact forest patches; in the inland zones, the fall was from 75 to 38%, between 1973 and 2014. The landscape-scale study reported in Kivinen et al. (2012) found decreased patch sizes and increased isolation of old-growth forests stands, coinciding with the increased dominance of young forests. This type of fragmentation reduces source habitats for pendulous lichens that can colonise neighbouring stands, with negative impacts on persistence due to the limited dispersal abilities of lichens (see Dettki et al. 2000).

### The impact of forestry on lichens

Here, I argue that the Swedish forestry model with the goal to maximise timber productivity has come to fundamentally shape boreal forests in Sweden, to the detriment of Sámi reindeer pastoralism. This argument is not new (Kivinen et al. 2010; Moen and Keskitalo 2010; Horstkotte et al. 2011; Korosuo et al. 2014; Sandström et al. 2016; Horstkotte and Moen 2019), and I do seek not to critique the goal of maximizing timber productivity per se, but rather to clarify the mechanisms through which specific forestry practices reduce the amount and distribution of lichens, and the main spatial and temporal scales at which this occurs.

The rapid loss of ground and pendulous lichens dates back to the intensive forest management regime that emerged with the industrialisation and mechanisation of forestry, and had become institutionalised in northern Sweden by the 1960s (Esseen et al. 1997; Östlund et al. 1997; Lundmark et al. 2013), and even earlier in some areas (Lundmark 2020). This regime emerged from models developed in Germany, and was argued to be best-suited for the unproductive climatic and environmental conditions of northern Sweden (*ibid.*). It was also driven by a growing timber demand from other industrialised countries in Europe, and was actively supported by the Swedish state (*ibid.*). It was accompanied by a rapid expansion of sawmills and pulp mills, in the context of a broader industrialisation process across northern Sweden (Östlund et al. 1997; Lundmark 2020).

In order to maximise timber productivity throughout stand succession, forestry in Sweden applies silvicultural methods such as clear-cutting, site preparation, dense reforestation, fertilisation, short rotation times, leaving logging residues and fire prevention practices and lodgepole pine plantation (Kivinen et al. 2010; Lindahl et al. 2017). These methods have contributed to the 50% increase in the standing volume of all tree species in productive forest land across Sweden identified between 1955 and 2018 (Swedish National Forestry Inventory 2021b). These methods impact lichen communities (and Sámi reindeer pastoralism) negatively through mechanisms that operate at different scales. These lead to, for example, changing light conditions, microclimates, soil moisture and nutrient amounts, and have been the subject of much research (e.g. Kivinen et al. 2010; for recent work on fertilisation see Jacobson et al. 2020).

**Fig. 3:**
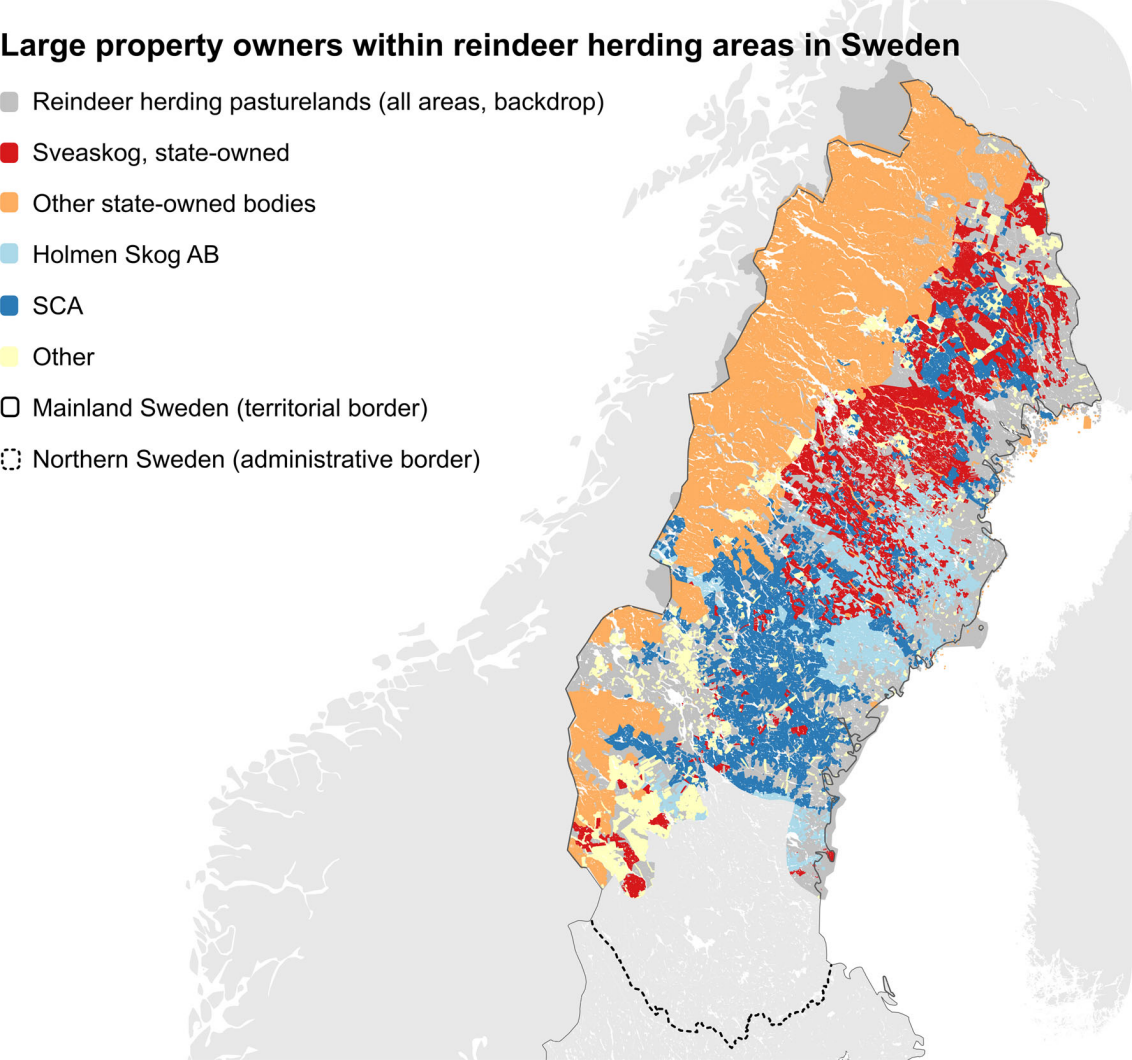
Map of large forest property owners in Sweden within reindeer herding areas. Data last updated 2015 but provide enough information to get an overview at the national scale (Sámediggi 2020, pp. 38–39)

The Swedish forestry model has been labelled as ‘more of everything’, and is legitimised through a utilitarian-economic rationality (Lindahl et al. 2017). It is further supported by national policies that frame trade-offs between economic development and environmental conservation as possible to resolve through technological solutions, i.e. ecological modernisation (*ibid.*). Market dynamics within a globalised capitalist market economy (e.g. surviving international competition) push timber productivity and profitability as overarching goals in firm-level decision-making, infringed on by legal frameworks, ownership directives and, more recently, certification systems. About 60% of land categorised as forests that lies within RHCs is owned by either large private corporations, the state/public, or the church (see Fig. 3). In order to optimise stand succession for timber productivity, forestry in these areas has been planned and managed on a stand-by-stand basis (i.e. the spatial scale of patch sizes) within rotation cycles (i.e. the temporal scale determined by the rotation time of timber harvesting) (see also Moen and Keskitalo 2010). To concretise these two scales: the mean size of individual harvested forest stands (“area of forest notified for final felling”) in productive forest land located in northern Sweden between 1995 and 2020 was 4.5 ha for individual owners, and 10.5 for other publicly or privately owned entities (Swedish National Forestry Inventory 2021c), and the shortest possible rotation cycle ranges from 80 to 120 years (Swedish Forestry Agency 2014; Horstkotte et al. 2016).

## The flux of nature: Non-equilibrium theory and grazing dynamics

Non-equilibrium theory is captured in the metaphor of the flux in nature. The non-equilibrium paradigm assumes that abiotic patterns are stochastic and variable; plant–herbivore interactions are weakly coupled and have abiotic drivers; population patterns are density independent and highly dynamic carrying capacities limit animal population tracking, and community/ecosystem characteristics have external drivers and are not primarily expressed through competition (Ellis and Swift 1988; Briske et al. 2003).

I now turn to the impacts on reindeer grazing dynamics of snow conditions, the cumulative effects of competing land uses and predator pressure. Snow conditions in particular are largely determined by climatic stochasticity, and although the literature that examined Sweden made no explicit reference to non-equilibrium theory, the model of functional resource heterogeneity (Fynn 2012) can be used to clarify how lichens fulfil different functions in the Sámi pastoral landscape, and highlight certain abiotic and biotic factors that shape their accessibility.

### Reindeer and functional resource heterogeneity

Fynn’s (2012) survey of rangeland ecology found that the stabilising properties of herbivore populations were “related to the ability of herbivores to move and access key functional vegetation resources” (p. 320). He argued that adaptive foraging in relation to functionally heterogeneous resources was “critical for free-ranging herbivores to maintain their body stores at stable and productive levels” (*ibid.* p. 321). Although the ability to store energy and nutrients during periods of forage resource abundance is recognised as important, it is understood to be insufficient for herbivores to survive extended periods of forage resource scarcity without significant casualties (ibid.; see also Ellis and Swift 1988). Therefore, the availability of these *key forage resources* during the most critical period has been determined as key for herbivore survival, and their availability is considered to be largely determined by stochastic, and both spatially and temporally variable weather event-driven dynamics (Fynn 2012).

In the context of Sámi reindeer pastoralism, Behnke (2000) argued that lichens were such key forage resources, and that the snow cover period (hereafter including ice) profoundly shaped reindeer–vegetation interactions. In Sweden, the interactions identified in earlier work by Fynn (2012) and Behnke (2000) can be exemplified in more recent case study research (Rosqvist et al. 2021) that combines weather data, GPS tracking of reindeer and Sámi pastoralist knowledge. The latter study shows that reindeer grazing patterns during the snow cover period are strongly determined by (sudden) changes in weather (especially in autumn and early-winter), which create snow conditions that reduce lichen accessibility (Rosqvist et al. 2021). At the same time, however, the Sámi pastoral landscape has been significantly transformed by human interventions. Consequently, the ability of reindeer to move and access lichens is acknowledged to be profoundly affected by anthropogenic factors as well (*ibid*.). I address this point in detail below.

### Abiotic factors: Snow condition and the functions of lichens

The main abiotic factors that affect lichen accessibility relate to snow conditions between *čakčadálvi* and *giđđa* (see Fig. 1C for definitions of the seasons). Although snow conditions also affect other functions in the Sámi pastoral landscape, not least migration pathways, this paper focuses on how they affect the accessibility of lichens for onsite reindeer.

Snow conditions (i.e. amount, density, hardness, wetness) are determined by variation in temperature, precipitation and wind, which, in turn, are driven by large-scale atmospheric and oceanic dynamics such as the North Atlantic Oscillation and the Arctic Oscillation (Callaghan et al. 2011; Bokhorst et al. 2016). However, local snow conditions are more immediately shaped by event-driven weather dynamics at much smaller temporal and spatial scales. Examples include ground temperature, exposure to sunlight and wind of individual locations (*ibid.*), and the occurrence and timing of rain-on-snow and freeze-and-thaw events (Rosqvist et al. 2021). Topographical features such as slopes in narrow ridges, and south-facing slopes tend to have softer snow, while large open areas exposed to wind tend to produce harder snow (Horstkotte and Roturier 2013; for a detailed analysis of snowpack dynamics in clear-cuts, see Schelker et al. 2013).

From a functional perspective, ground lichens represent a carbohydrate-rich forage resource, and provide reindeer with energy, especially during the demanding *dálvi* season (Heggberget et al. 2002). This function requires snow conditions that allow access to forage, captured in the word *guohtun* (Eira et al. 2013). The degree to which different snow conditions affect *guohtun* is well understood among Sámi pastoralists (*ibid.*). The most critical states are exceptionally deep snow, and extensive basal ice formations under snow (*ibid.*). The latter is created in late-autumn/early-winter, and they lock away ground lichens until spring, possibly even resulting in mould formations (*ibid.*; see also Rosqvist et al. 2021). Furthermore, a range of other snowpack characteristics such as snow hardness/depth and ice layer formations at different levels of the snowpack affect, for example, how much energy is required for cratering (Heggberget et al. 2002; Inga 2009; Roturier and Roué 2009; Eira et al. 2013).

Turning to pendulous lichens, these also represent carbohydrate-rich forage resources. However, they are more important from *dálvi* until the end of *giđđa*, and during periods with poor snow conditions, known as *goavvi* (Riseth et al. 2011). Pendulous lichens are important during *goavvi* because migrating to continuous old-growth forests where they exist represents the traditional adaptive response to when snow conditions limit the accessibility of ground lichens (Berg et al. 2011; Axelsson-Linkowski et al. 2020; Rosqvist et al. 2021). Pendulous lichens can be accessed if released to the ground by wind, manually by reindeer herders, or if the snow cover is deep and hard enough to support the weight of a reindeer, *ceavvi* (Riseth et al. 2011). But when reindeer sustain themselves on pendulous lichens, they may disperse over larger areas than when they can rely on ground lichens (Rosqvist et al. 2021).

### Biotic factors: Herd structures and grazing peace

Reindeer dig through snow to access ground lichens, which has an energy cost. Studies show that reindeer crater where the snow is, on average, less deep and hard within a feeding site, guided by their smell (Johnson et al. 2000). Cratering sites tend to be locations with the greatest ground lichen cover (Roturier and Bergsten 2006).

The distribution of sex and age within the herd are other important biotic factors that affect energy expenditures, cratering abilities and grazing patterns of individuals, groups and herds (Heggberget et al. 2002). The current dominant herd structure suggests that larger shares of females increase calf productivity, and slaughtering of males and calves in autumn reduces the mortality rate of the herd during winter (Uboni et al. 2020). This argument relies on the fact that does and calves require less energy than bucks (Åhman and White 2018), and that calves have the highest mortality rate during winter (Mattisson et al. 2014). Although bucks require more energy, they are better at cratering, and may exhibit fewer avoidance behaviours in relation human activity and infrastructure (Skum et al. 2016).

For reindeer to stay at a feeding site and graze effectively, they must not be exposed to repeated threats and disturbances, a factor referred to as *grazing peace*, which is negatively affected by human activity and infrastructure, along with predator pressure (Axelsson-Linkowski et al. 2020: p. 485; see also Sámediggi 2021a). Regarding human activity and infrastructure, the review by Skarin and Åhman (2014) highlighted that “reindeer exhibit avoidance behaviours up to 12 km away from infrastructure and sites of human activity and that the area they avoid may shift between seasons and years” (p. 1041). These so-called disturbance zones (i.e. areas that affect reindeer behaviour and cause avoidance behaviour) have been quantified by using GPS tracking, notably in relation to wind energy turbines (Skarin et al. 2018). Regarding predator pressure, mortality studies show correlations between predator pressure and herd size due to the presence of, for example, lynx, wolverine, brown bear and wolf populations in boreal forests (Åhman et al. 2014; Mattisson et al. 2014; Sivertsen et al. 2016; Aronsson and Persson 2017; Hobbs et al. 2019).

### Forage accessibility over time and space

**Fig. 4:**
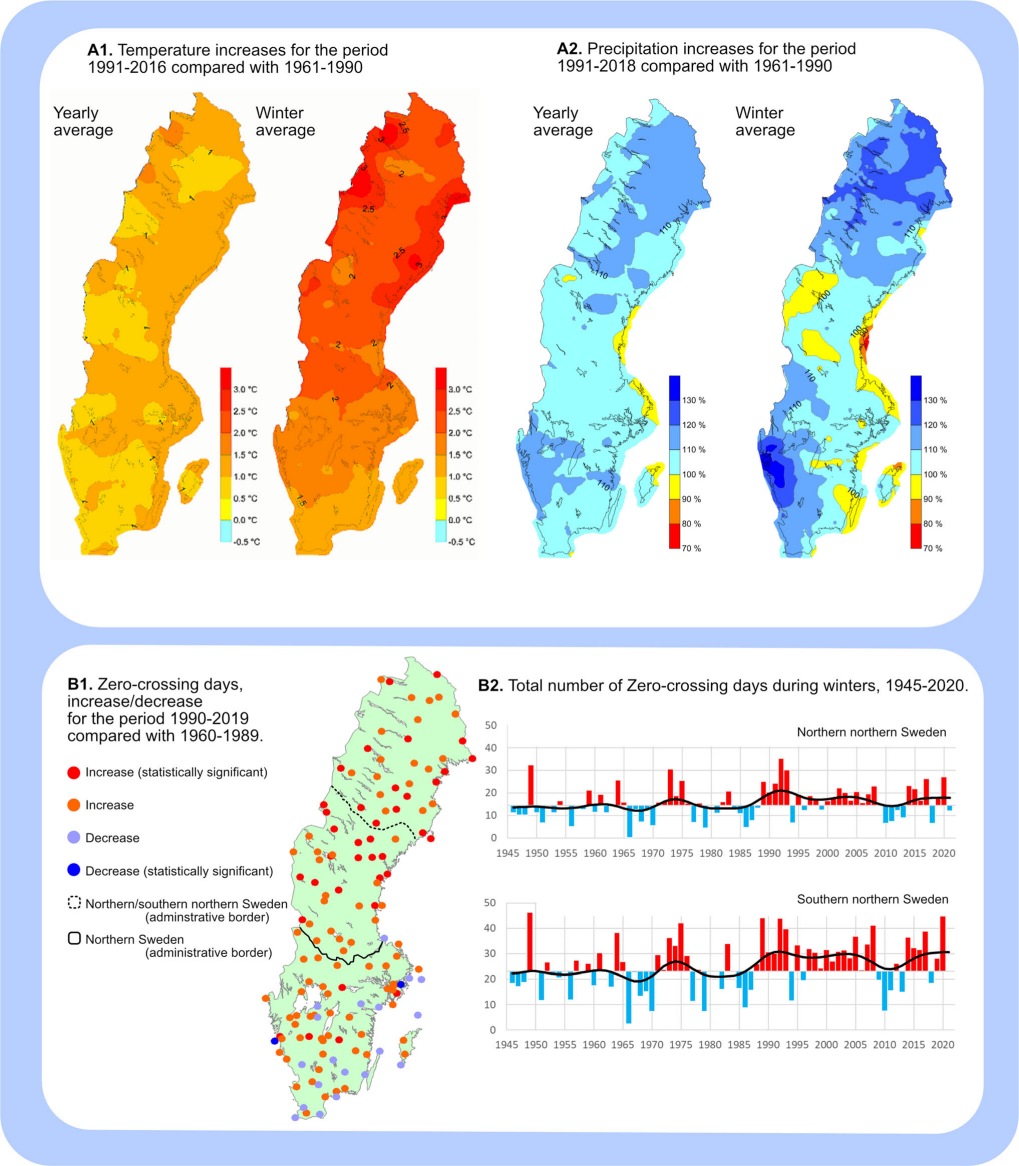
Climate data from SMHI. Winter in this data is defined as December, January and February. **A**: Temperature and precipitation increases (modified from SMHI 2021c). **B**: Zero-crossing days, increase/decrease and total number of during winters (modified from SMHI 2021b)

Based on the meteorological data from weather stations, we know that northern Sweden has experienced higher temperatures and increased precipitation in the past three decades due to climate change (SMHI 2021c; see Fig. 4A). We also know that the length of snow cover periods has decreased between 1949/1950 and 2019/2020 (SMHI 2021a), and that the amounts of precipitation during extreme precipitation events have increased between 1900 and 2011 (Wern 2012). Even though snow conditions are the outcome of complex causal networks with limited data availability, some studies have attempted to identify how conditions have changed over time in Sweden.

Snow data (i.e. grain size/compactness, layer hardness/dryness and depth) have been analysed over longer time periods at the Swedish Abisko Scientific Research Station, located in a mountainous area (Johansson et al. 2011). Utilising snow profile observations recorded every second week between 1961 and 2009, Johansson et al. (2011) found that the position of “very hard snow layers” had shifted to the lower part of the snowpack on twice as many occasions during the period 1993–2009 compared to 1961–1976 and 1977–1992; where it more significantly reduces access to ground lichens. Climate data on daily maximum and minimum temperatures and precipitation amounts have also been used to construct proxies for snow conditions. One example is the use of zero-crossing days as a proxy for ice crust formation (Lundqvist et al. 2009), and studies have found that they have increased in northern Sweden between 1945 and 2020, especially during winters (SMHI 2021b: see Fig. 4B).

Encroachment due to competing land uses can remove forage and limit its accessibility. Cumulative effects of encroachments have been analysed using a variety of sector-based economic, GIS and qualitative data (Kløcker-Larsen et al. 2016; Österlin and Raitio 2020; Fohringer et al. 2021). Österlin and Ratio’s (2020) macro-level study of RHCs identified an increase in permits granted for mining (from 1000 ha to over 20 000 ha between 1960 and 2017) and wind turbines (from 48 to 983 operating turbines between 2003 and 2017). Fohringer et al. (2021) focussed on an individual mountain RHC, and their case study concluded that 34% of pasturelands had become functionally unavailable; this can be compared to the case study reported by Kløcker-Larsen et al. (2016), which concluded that 54% of winter pasturelands were within aforementioned disturbance zones. Regarding predator pressure, the Sámi parliament recently reported that, for many RHCs, 20–40% of the herd (mainly calves) was killed by predators annually, which is much higher than the 10% tolerance level defined by the state (Sámediggi 2021b).

### Navigating flux when under a high degree of stress

Here, I argue that Sámi reindeer pastoralism with the goal to maintain natural grazing-based practices is under a high degree of stress during the snow cover periods. This is not a new argument (Moen and Keskitalo 2010; Furberg et al. 2011; Löf 2013; Axelsson-Linkowski et al. 2020), and I do not seek to discuss the goal of maintaining natural grazing-based responses to poor snow conditions per se, but rather to clarify the implications of changing ecological conditions for Sámi pastoral knowledge and practices with such a goal in mind.

The many practices that surround Sámi reindeer pastoralism have become more labour intensive, with negative impacts on opportunities to make a good living and detrimental effects on well-being (Axelsson-Linkowski et al. 2020; Österlin and Raitio 2020). This is partly due to RHCs having to spend more of their limited resources on consultations with other land users to protect and improve landscape functions, in a complicated web of regulations that “can, even at best, only deliver the status quo” (Österlin and Raitio 2020: p. 20; see also Buchanan et al. 2016). But another important factor is how changes in ecological conditions challenge traditional knowledge and practices. As Axelsson-Linkowski et al. (2020) noted, the “rapidly changing environmental circumstances are forcing herders into uncharted territories where […] traditional strategies and the transmission of knowledge between generations may be of limited use” (p. 481; see also Furberg et al. 2018).

At the present time, Sámi pastoralists in Sweden increasingly rely on capital inputs associated with increased costs and material throughput to cope with poor grazing conditions during snow cover periods (Axelsson-Linkowski et al. 2020; Uboni et al. 2020; Rosqvist et al. 2021). One example is the increase in the usage of vehicles. For instance, helicopters may be used to locate and gather widely dispersed reindeer herds, or trucks may be used to move trapped reindeer to suitable pastures (Rosqvist et al. 2021). Another example is the increase in the usage of supplementary feeding in corrals, or when free-ranging (Brännlund and Axelsson 2011; Uboni et al. 2020; Horstkotte et al. 2021). This development is illustrated by the increase in annual sales of factory-made (grain-based) reindeer feed from the two main producers. Sales have increased to over 30 kg per reindeer per year by 2015 since its introduction in 1985, with spikes up to 60 kg during exceptionally bad winters (Uboni et al. 2020). But many pastoralists consider that the increased reliance on supplementary feeding, and the stationary practices that goes hand-in-hand with it are undesirable for several reasons. These include worsening reindeer health due to an increased risk of disease; more domesticated behaviours among reindeer; negative impacts on vegetation and soils; threats to grazing rights as Sámi pastoralists must continue to use the land in order to maintain their rights; and that a regime shift towards increased supplementary feeding, ultimately, represents a break away from traditional Sámi reindeer pastoralism (Horstkotte et al. 2021).

## Implications

My literature review focused on the ecological dynamics that limit the availability of lichens as reindeer forage during snow cover periods. The findings suggest the following about the *status-quo* of Sámi pastoral landscapes in Sweden.

In Sweden, forestry has (with significant spatial coverage) profoundly altered biotic and abiotic structures and processes in boreal forests. The removal, degradation and fragmentation of lichen habitats have decreased the availability of lichens that are key forage resources for reindeer. At the same time, climate change is impacting snow conditions (also with significant spatial coverage) and this, together with the cumulative effects of competing land uses and predator pressure (both with larger regional differences), means that Sámi reindeer pastoralism is becoming more labour- and capital-intensive, and its traditional knowledge systems and adaptive responses are being challenged. Sámi pastoralists may cope with poor grazing conditions during snow cover periods by increasing their mobility and relying on supplementary feeding to limit reindeer mortality. Although some general trends are seen across all areas where Sámi reindeer pastoralism is practiced, many of the impacts of these multiple stressors are unevenly distributed, which means that each RHC is affected differently.

Immanent critique identifies internal gaps and limitations in existing theories and practices, and uses them to articulate alternatives that can resolve tensions in relation to a concrete phenomenon. To structure my discussion on such alternatives, I draw on the concept of *intervention ecology*, which, in broad terms, argues that applied ecology can help in developing interventions that could benefit both humanity and other organisms (Hobbs et al. 2011). Given that the world is characterised by rapid environmental change and ecosystems that are in flux, notions of *restoration* and *conservation* can be associated with moral hazards (*ibid.*). Hobbs et al. (2011) instead argue for the elaboration of goal-driven interventions that seek to not only alter the biotic and abiotic structures and processes found within ecosystems and landscapes, but also address social and policy settings. While, in general, interventions need to target different (social–)ecological scales depending on the goal, those that link to leverage points that support feedback loops which maintain a state are particularly important (*ibid.*). In this context, I use immanent critique to address the question of when equilibrium and non-equilibrium dynamics apply, and at what scales.

Equilibrium theory, specifically successional theory, is relevant to explain vegetation dynamics within the Sámi pastoral landscape in relation to forestry. It suggests that as long as the form and distribution of disturbances caused by the Swedish forestry model do not change, ground and pendulous lichen habitats will continue to be limited and fragmented—a consequence of the forest management and planning practices of large forest owners in particular. Empirically informed arguments can demonstrate the successional pathways that follow structured forestry practices. For example, clear-cutting that is followed by intense site preparation with fertiliser may promote timber productivity; however, this is at the expense of lichen habitat formation and sustenance. Such arguments can also suggest alternative forest management and planning practices that could lead in other directions, notably successional pathways that are more conducive to lichen habitat formation. However, equilibrium theory is not the best approach to analyse Sámi reindeer pastoralism, not least because it can legitimise overstocking arguments when forage availability is limited by factors other than the herbivore population. Moreover, it can uncritically accept that increased labour and capital intensity (i.e. work, fuel and feed) are desirable ways to control stochasticity.

Non-equilibrium theory (e.g. functional resource heterogeneity) can help to explain how climatic stochasticity shapes reindeer grazing patterns in pastoral landscapes. It provides an insight into the critical role of lichens in Sámi reindeer pastoralism, and how lichen accessibility is profoundly shaped by snow conditions that are governed by stochastic and variable temperature and precipitation patterns. Furthermore, it helps in understanding how the impacts of snow conditions are shaped by the general state of seasonal pasturelands located in boreal forests, as well as specific weather events at individual locations (in time and space) within those pasturelands, especially during the early snow cover period. It clarifies how natural grazing-based Sámi reindeer pastoralism depends on pastoralists’ ability to maintain spatial and temporal flexibility, and how this, in turn, relies on multiple functions in different seasonal pasturelands. On the other hand, this conceptualisation does not help in understanding how disturbances affect vegetation dynamics in lichen habitats across boreal forests—which is no less important in informing interventions intended to improve the situation.

I argue that forestry and other land uses could support Sámi reindeer pastoralism by adopting relevant goals, management and planning practices—and that not doing so is a decision that also has implications. In particular, the problem of the reduced availability of forage during snow cover periods could be more comprehensibly understood and addressed if sustainable land use planning at local, regional and even national levels adopted the Sámi pastoral landscape perspectives of RHCs, and focussed on incorporating the multiple ecological scales identified in the two aforementioned overlapping ecological dynamics into decision-making processes. The academic literature has already put forward many concrete suggestions on how lichen habitat formation and sustenance could be promoted at the scale of forest stands (increasing rotation time, reducing soil disturbance, leaving larger groups of trees, considering efficient dispersal distances, more vigorous early thinning, lichen transplantation) and at the landscape scale (preserving large continuous old-growth forest and increasing the area and connectivity of non-use forest areas) (Kivinen et al. 2010; Roturier et al. 2011; Korosuo et al. 2014; Horstkotte et al. 2016; St John et al. 2016).

But better knowledge does not, by itself, lead to better practice in society, demonstrated by the fact that many of the proposed recommendations are not new. The ecological argument that is developed in this article clarifies how political tension that are rooted in society emerge as intervention ecologies are to be developed. Specifically, increasing the availability of reindeer forage during snow cover periods requires certain degree of infringement on the land use rights of primarily large forest property owners. Changes are needed to both planning and management that likely would reduce timber output, which, in turn, challenges the state’s approach to ecological modernisation. Although a multi-purpose intervention ecology could incorporate more non-economic values into sustainable land use planning, it cannot be implemented without overcoming political resistance that is rooted in material interests and social relations.

## A concluding discussion

It is clear that in Sweden, forestry and Sámi reindeer pastoralism have fundamentally different goals, scales and ecological understandings. Any dialogue between these two broad actor constellations is bound to be characterised by conceptual difficulties and conflicts of interest. In practice, this is just the tip of the iceberg, as even more interests are enmeshed in these social relations due to the number of other land uses and actors involved, leading to political dilemmas often characterised as trade-offs.

Sámi pastoralists are part of the indigenous Sámi people in Sweden, or Swedish Sápmi, and have been subject to a long history of misrecognition and maldistribution (e.g. Persson et al. 2017). As Sámi pastoralists are not viewed by the government as holding property rights to their pasturelands (although recent legal case developments may change that interpretation, see Allard and Brännström 2021), their political capacity to shape the decisions of forest owners within a capitalist market economy is limited to actions in the public sphere. In the present situation, it appears that large property owners must be compelled to give up some of their land use rights, through some form of landscape-by-landscape planning. However, Sámi pastoralists will likely need allies in political processes if the type of intervention ecology advocated in this article is to be implemented.

The conceptualisation presented in the present article could be used to articulate a new, multi-purpose forest intervention ecology. Such an approach could support outcomes that meet goals other than timber productivity and that people could mobilize for achieving (Boda et al. 2021; Harnesk and Isgren 2021; see also Visseren-Hamakers et al. 2021). Goals could include sustainable conditions for natural grazing-based responses to poor snow conditions for Sámi reindeer pastoralism; biodiversity conservation; near-term carbon sequestration; and a shift in production towards long-lived timber products. But to mobilize broader support, any intervention ecology must likely also address broader social inequalities that exist prior to and throughout any transition process, such as urban–rural divides. While that conversation belongs to the realm of politics, the conceptualisation developed in the present article (and other research) can help inform the creation of political collectives based on common interests, values and identities rooted in (social–)ecological perspectives—a type of agency needed in the current context of multiple and intersecting ecological crises and social inequalities that require transformational change.

## Supplementary Information

Below is the link to the electronic supplementary material.

Supplementary file1 (PDF 1004 kb)
