# Supplementary material for: The decreasing availability of reindeer forage in boreal forests during snow cover periods: A Sámi pastoral landscape perspective in Sweden
Source: Ambio. 2022 Jun 21;51(12):2508–23. doi: 10.1007/s13280-022-01752-w (PMC9583999; doi:10.1007/s13280-022-01752-w)
Supplement: Supplementary file 1 — Supplementary file1 (PDF 1004 kb) [file 13280_2022_1752_MOESM1_ESM.pdf]

Ambio

Supplementary Information.

This supplementary information has not been peer reviewed.

Title: The decreasing availability of reindeer forage in boreal forests during snow cover periods – a Sámi pastoral landscape perspective in Sweden

Literature directly incorporated from the Web of Science and Scopus

Total number of sources: 34

Åhman, B., Svensson, K., & Rönnegård, L. (2014). High Female Mortality Resulting in Herd Collapse in Free-Ranging Domesticated Reindeer (Rangifer tarandus tarandus) in Sweden. *PLoS ONE*, 9(10), e111509.

Aronsson, M., & Persson, J. (2017). Mismatch between goals and the scale of actions constrains adaptive carnivore management: the case of the wolverine in Sweden. *Animal Conservation*, 20(3), 261–269.

Axelsson-Linkowski, W., Fjellström, A. M., Sandström, C., Westin, A., Östlund, L., & Moen, J. (2020). Shifting Strategies between Generations in Sami Reindeer Husbandry: the Challenges of Maintaining Traditions while Adapting to a Changing Context. *Human Ecology*, 48(4), 481–490.

Berg, A., Josefsson, T., & Östlund, L. (2011). Cutting of lichen trees: A survival strategy used before the 20th century in northern Sweden. *Vegetation History and Archaeobotany*, 20(2), 125–133.

Berg, A., Östlund, L., Moen, J., & Olofsson, J. (2008). A century of logging and forestry in a reindeer herding area in northern Sweden. *Forest Ecology and Management*, 256(5), 1009–1020.

Callaghan, T. V., Johansson, M., Brown, R. D., Groisman, P. Y., Labba, N., Radionov, V., Barry, R. G., Bulygina, O. N., Essery, R. L. H., Frolov, D. M., Golubev, V. N., Grenfell, T. C., Petrushina, M. N., Razuvaev, V. N., Robinson, D. A., Romanov, P., Shindell, D., Shmakin, A. B., Sokratov, S. A., … Yang, D. (2011). The changing face of arctic snow-cover: A synthesis of observed and projected changes. *Ambio*, 40(S1), 17–31.

Cogos, S., Östlund, L., & Roturier, S. (2019). Forest Fire and Indigenous Sami Land Use: Place Names, Fire Dynamics, and Ecosystem Change in Northern Scandinavia. *Human Ecology*, 47(1), 51–64.

Dettki, H., & Esseen, P. A. (1998). Epiphytic macrolichens in managed and natural forest landscapes: A comparison at two spatial scales. *Ecography*, 21(6), 613–624.

Dettki, Holger, & Esseen, P. A. (2003). Modelling long-term effects of forest management on epiphytic lichens in northern Sweden. *Forest Ecology and Management*, 175(1–3), 223–238.

Esseen, P.-A., Ekström, M., Westerlund, B., Palmqvist, K., Jonsson, B. G., Grafström, A., & Ståhl, G. (2016). Broad-scale distribution of epiphytic hair lichens correlates more with climate and nitrogen deposition than with forest structure. *Canadian Journal of Forest Research*, 46(11), 1348–1358.

Esseen, P. A., Renhorn, K. E., & Pettersson, R. B. (1996). Epiphytic lichen biomass in managed and old-growth boreal forests: Effect of branch quality. *Ecological Applications*, 6(1), 228–238.

Furberg, M., Hondula, D. M., Saha, M. V., & Nilsson, M. (2018). In the light of change: a mixed methods investigation of climate perceptions and the instrumental record in northern Sweden. *Population and Environment*, 40(1), 47–71.

Hörnberg, G., Josefsson, T., DeLuca, T. H., Higuera, P. E., Liedgren, L., Östlund, L., & Bergman, I. (2018). Anthropogenic use of fire led to degraded scots pine-lichen forest in northern Sweden. *Anthropocene*, 24, 14–29.

Horstkotte, T., Utsi, T. A., Larsson-Blind, Å., Burgess, P., Johansen, B., Käyhkö, J., Oksanen, L., & Forbes, B. C. (2017). Human-animal agency in reindeer management: Sámi herders' perspectives on vegetation dynamics under climate change. *Ecosphere*, 8(9), e01931.

Horstkotte, Tim, & Moen, J. (2019). Successional pathways of terrestrial lichens in changing Swedish boreal forests. *Forest Ecology and Management*, 453, 117572.

Horstkotte, Tim, Moen, J., Lämås, T., & Helle, T. (2011). The Legacy of Logging—Estimating Arboreal Lichen Occurrence in a Boreal Multiple-Use Landscape on a Two Century Scale. *PLoS ONE*, 6(12), e28779.

Horstkotte, Tim, & Roturier, S. (2013). Does forest stand structure impact the dynamics of snow on winter grazing grounds of reindeer (Rangifer t. tarandus)? *Forest Ecology and Management*, 291, 162–171.

Horstkotte, Tim, Sandström, C., & Moen, J. (2014). Exploring the multiple use of boreal landscapes in Northern Sweden: The importance of social-ecological diversity for mobility and flexibility. *Human Ecolog*y, 42(5), 671–682.

Jacobson, S., Högbom, L., & Ring, E. (2020). Long-term responses of understory vegetation in boreal Scots pine stands after nitrogen fertilization. *Scandinavian Journal of Forest Research,* 35(3–4), 139–146.

Jonsson, B. G., Dahlgren, J., Ekström, M., Esseen, P.-A., Grafström, A., Ståhl, G., & Westerlund, B. (2021). Rapid Changes in Ground Vegetation of Mature Boreal Forests—An Analysis of Swedish National Forest Inventory Data. *Forests*, 12(4), 475.

Jonsson Čabrajič, A. V., Moen, J., & Palmqvist, K. (2010). Predicting growth of mat-forming lichens on a landscape scale - comparing models with different complexities. *Ecography*, 33(5), 949–960.

Kivinen, S., Berg, A., Moen, J., Östlund, L., & Olofsson, J. (2012). Forest fragmentation and landscape transformation in a reindeer husbandry area in Sweden. *Environmental Management*, 49(2), 295–304.

Kivinen, S., Moen, J., Berg, A., & Eriksson, Å. (2010). Effects of modern forest management on winter grazing resources for reindeer in Sweden. *Ambio*, 39(4), 269–278.

Korosuo, A., Sandström, P., Öhman, K., & Eriksson, L. O. (2014). Impacts of different forest management scenarios on forestry and reindeer husbandry. *Scandinavian Journal of Forest Research,* 29(sup1), 234–251.

Österlin, C., & Raitio, K. (2020). Fragmented Landscapes and Planscapes—The Double Pressure of Increasing Natural Resource Exploitation on Indigenous Sámi Lands in Northern Sweden. *Resources*, 9(9), 104.

Rosqvist, G. C., Inga, N., & Eriksson, P. (2021). Impacts of climate warming on reindeer herding require new land-use strategies. *Ambio*, 1–16.

Roturier, S., & Bergsten, U. (2006). Influence of soil scarification on reindeer foraging and damage to planted Pinus sylvestris seedlings. *Scandinavian Journal of Forest Research*, 21(3), 209–220.

Roturier, S., & Roué, M. (2009). Of forest, snow and lichen: Sámi reindeer herders' knowledge of winter pastures in northern Sweden. *Forest Ecology and Management*, 258(9), 1960–1967.

Sandström, P., Cory, N., Svensson, J., Hedenås, H., Jougda, L., & Borchert, N. (2016). On the decline of ground lichen forests in the Swedish boreal landscape: Implications for reindeer husbandry and sustainable forest management. *Ambio*, 45(4), 415–429.

Sandström, P., Granqvist Pahlén, T., Edenius, L., Tømmervik, H., Hagner, O., Hemberg, L., Olsson, H., Baer, K., Stenlund, T., Göran Brandt, L., & Egberth, M. (2003). Conflict Resolution by Participatory Management: Remote Sensing and GIS as Tools for Communicating Land-use Needs for Reindeer Herding in Northern Sweden. *Ambio*, 32(8), 557–567.

Schelker, J., Kuglerová, L., Eklöf, K., Bishop, K., & Laudon, H. (2013). Hydrological effects of clear-cutting in a boreal forest - Snowpack dynamics, snowmelt and streamflow responses. *Journal of Hydrology*, 484, 105–114.

St John, R., Öhman, K., Tóth, S. F., Sandström, P., Korosuo, A., & Eriksson, L. O. (2016). Combining spatiotemporal corridor design for reindeer migration with harvest scheduling in Northern Sweden. *Scandinavian Journal of Forest Research*, 31(7), 655–663.

Uboni, A., Åhman, B., & Moen, J. (2020). Can management buffer pasture loss and fragmentation for Sami reindeer herding in Sweden? *Pastoralism*, 10(1), 23.

Uboni, A., Blochel, A., Kodnik, D., & Moen, J. (2019). Modelling occurrence and status of mat-forming lichens in boreal forests to assess the past and current quality of reindeer winter pastures. *Ecological Indicators*, 96, 99–106.

Additional sources - including articles, statistics, and GIS data

Total number of sources: 54

Åhman, B., & White, R. G. (2018). Rangifer diet and nutritional needs. In M. Tryland & S. J. Kutz (Eds.), *Reindeer and caribou: Health and disease* (pp. 107–134). Taylor & Francis.

Bokhorst, S., Pedersen, S. H., Brucker, L., Anisimov, O., Bjerke, J. W., Brown, R. D., Ehrich, D., Essery, R. L. H., Heilig, A., Ingvander, S., Johansson, C., Johansson, M., Jónsdóttir, I. S., Inga, N., Luojus, K., Macelloni, G., Mariash, H., McLennan, D., Rosqvist, G. N., … Callaghan, T. V. (2016). Changing Arctic snow-cover: A review of recent developments and assessment of future needs for observations, modelling, and impacts. *Ambio* 45(5), 516–537.

Benjaminsen, Tor A., Reinert, H., Sjaastad, E., & Sara, M. N. (2015). Misreading the Arctic landscape: A political ecology of reindeer, carrying capacities, and overstocking in Finnmark, Norway. Norsk Geografisk Tidsskrift - Norwegian Journal of Geography, 69(4), 219–229.

Boudreault, C., Bergeron, Y., & Coxson, D. (2009). Factors controlling epiphytic lichen biomass during postfire succession in black spruce boreal forests. *Canadian Journal of Forest Research*, 39(11), 2168–2179.

Brännlund, I., & Axelsson, P. (2011). Reindeer management during the colonization of Sami lands: A long-term perspective of vulnerability and adaptation strategies. *Global Environmental Change*, 21(3), 1095–1105.

Buchanan, A., Reed, M. G., & Lidestav, G. (2016). What's counted as a reindeer herder? Gender and the adaptive capacity of Sami reindeer herding communities in Sweden. *Ambio*, 45(S3), 352–362.

Coxson, D. S., & Coyle, M. (2003). Niche partitioning and photosynthetic response of alectorioid lichens from subalpine spruce-fir forest in north-central British Columbia, Canada: The role of canopy microclimate gradients. *Lichenologist*, 35(2), 157–175.

Dettki, H., Klintberg, P., & Esseen, P. A. (2000). Are epiphytic lichens in young forests limited by local dispersal? *Ecoscience*, 7(3), 317–325.

Eira, I. M. G., Jaedicke, C., Magga, O. H., Maynard, N. G., Vikhamar-Schuler, D., & Mathiesen, S. D. (2013). Traditional Sámi snow terminology and physical snow classification-Two ways of knowing. *Cold Regions Science and Technology*, 85, 117–130.

Esseen, Per-Anders. (2019). Strong influence of landscape structure on hair lichens in boreal forest canopies. *Canadian Journal of Forest Research*, 49(8), 994–1003.

Esseen, Per-Anders, Ehnström, B., Ericson, L., & Sjöberg, K. (1997). Boreal Forests. *Ecological Bulletins*, 46, 16–47.

Fohringer, C., Rosqvist, G., Inga, N., & Singh, N. J. (2021). Reindeer husbandry in peril?—How extractive industries exert multiple pressures on an Arctic pastoral ecosystem. *People and Nature*, 3(4), 872–886.

Furberg, M., Evengård, B., & Nilsson, M. (2011). Facing the limit of resilience: perceptions of climate change among reindeer herding Sami in Sweden. *Global Health Action*, 4(1), 8417.

Gaio-Oliveira, G., Moen, J., Danell, Ö., & Palmqvist, K. (2006). Effect of simulated reindeer grazing on the re-growth capacity of mat-forming lichens. *Basic and Applied Ecology*, 7(2), 109–121.

Heggberget, T. M., Gaare, E., & Ball, J. P. (2002). Reindeer (Rangifer tarandus) and climate change: Importance of winter forage. *Rangifer*, 22(1), 13.

Hobbs, N. T., Andrén, H., Persson, J., Aronsson, M., Hobbs, N. T., Andrén, H., Persson, J., Aronsson, M., & Chapron, G. (2019). Native predators reduce harvest of reindeer by Sámi pastoralists. *Ecological Applications*, 22(5), 1640–1654.

Horstkotte, Tim, Lépy, È., Risvoll, C., et al. (2021). *Supplementary feeding in reindeer husbandry. Results from a workshop with reindeer herders and researchers from Norway, Sweden and Finland.* Umeå University.

Horstkotte, Tim, Lind, T., & Moen, J. (2016). Quantifying the Implications of Different Land Users' Priorities in the Management of Boreal Multiple-Use Forests. *Environmental Management*, 57(4), 770–783.

Inga, B. (2009). Reindeer (Rangifer tarandus tarandus) feeding on lichens and mushrooms: traditional ecological knowledge among reindeer-herding Sami in northern Sweden. *Rangifer*, 27(2), 93.

Jaakkola, L. M., Helle, T. P., Soppela, J., Kuitunen, M. T., & Yrjönen, M. J. (2006). Effects of forest characteristics on the abundance of alectorioid lichens in northern Finland. *Canadian Journal of Forest Research*, 36(11), 2955–2965.

Jacobs, S., Martín-López, B., Barton, D. N., Dunford, R., Harrison, P. A., Kelemen, E., Saarikoski, H., Termansen, M., García-Llorente, M., Gómez-Baggethun, E., Kopperoinen, L., Luque, S., Palomo, I., Priess, J. A., Rusch, G. M., Tenerelli, P., Turkelboom, F., Demeyer, R., Hauck, J., … Smith, R. (2018). The means determine the end – Pursuing integrated valuation in practice. *Ecosystem Services*, 29, 515–528.

Johansson, C., Pohjola, V. A., Jonasson, C., & Callaghan, T. V. (2011). Multi-decadal changes in snow characteristics in sub-Arctic Sweden. *Ambio*, 40(6), 566–574.

Johnson, C. J., Parker, K. L., & Heard, D. C. (2000). Feeding site selection by woodland caribou in north-central British Columbia. *Rangifer*, 20(5), 158.

Jougda, L. (2017). *Renbruksplan. Manual för beteslandsindelning - version mars 2017*. Sámediggi.

Kløcker-Larsen, R., Raitio, K., Sandström, P., Skarin, A., Stinnerbom, M., Wik-Karlsson, J., Sandström, S., Österlin, C., & Buhot, Y. (2016). Kumulativa effekter av exploateringar på renskötseln: Vad behöver göras inom tillståndsprocesser (Rapport 6722). *Naturvårdsverket*: Stockholm, Sweden.

Lindahl, K. B., Sténs, A., Sandström, C., Johansson, J., Lidskog, R., Ranius, T., & Roberge, J. M. (2017). The Swedish forestry model: More of everything? *Forest Policy and Economics,* 77, 44–55.

Löf, A. (2013). Examining limits and barriers to climate change adaptation in an Indigenous reindeer herding community. *Climate and Development*, 5(4), 328–339.

Lundmark, H. (2020). *Clear-cutting - The most discussed logging method in Swedish forest history*. Dissertation. Swedish University of Agricultural Sciences.

Lundmark, H., Josefsson, T., & Östlund, L. (2013). The history of clear-cutting in northern Sweden - Driving forces and myths in boreal silviculture. *Forest Ecology and Management*, 307, 112–122.

Lundqvist, H., Norell, L., & Danell, Ö. (2009). Multivariate characterisation of environmental conditions for reindeer husbandry in Sweden. *Rangifer*, 27(1).

Mattisson, J., Arntsen, G. B., Nilsen, E. B., Loe, L. E., Linnell, J. D. C., Odden, J., Persson, J., & Andrén, H. (2014). Lynx predation on semi-domestic reindeer: Do age and sex matter? *Journal of Zoology*, 292(1), 56–63.

Moen, J., & Keskitalo, E. C. H. (2010). Interlocking panarchies in multi-use boreal forests in Sweden. *Ecology and Society*, 15(3).

Nash, T. H. (2008). *Lichen biology (Second Edition)*. Cambridge University Press.

Östlund, L., Zackrisson, O., & Axelsson, A. L. (1997). The history and transformation of a Scandinavian boreal forest landscape since the 19th century. *Canadian Journal of Forest Research*, 27(8), 1198–1206.

Riseth, J. Å., Tømmervik, H., Helander-Renvall, E., Labba, N., Johansson, C., Malnes, E., Bjerke, J. W., Jonsson, C., Pohjola, V., Sarri, L. E., Schanche, A., & Callaghan, T. V. (2011). Sámi traditional ecological knowledge as a guide to science: Snow, ice and reindeer pasture facing climate change. *Polar Record*, 47(3), 202–217.

Riseth, Tømmervik, H., & Bjerke, J. W. (2016). 175 years of adaptation: North Scandinavian Sámi reindeer herding between government policies and winter climate variability (1835–2010). *Journal of Forest Economics*, 24, 186–204.

Roturier, S., Sundén, M., & Bergsten, U. (2011). Re-establishment rate of reindeer lichen species following conventional disc trenching and HuMinMix soil preparation in Pinus -lichen clear-cut stands: a survey study in northern Sweden. *Scandinavian Journal of Forest Research*, 26(2), 90–98.

Sámediggi (2021a). *Rennäring*. https://www.sametinget.se/rennaring [accessed 2022-02-28]

Sámediggi (2021b). *Årsredovisning*. Sámediggi: Kiruna, Sweden.

Sillett, S. C., & Antoine, M. E. (2004). Lichens and Bryophytes in Forest Canopies. In Lowman, M. D. & Rinker, B. (Eds). *Forest Canopies: Second Edition* (pp. 151–174). Elsevier.

Sivertsen, T. R., Åhman, B., Steyaert, S. M. J. G., Rönnegård, L., Frank, J., Segerström, P., Støen, O., & Skarin, A. (2016). Reindeer habitat selection under the risk of brown bear predation during calving season. *Ecosphere*, 7(11), e01583.

Skarin, A., & Åhman, B. (2014). Do human activity and infrastructure disturb domesticated reindeer? The need for the reindeer's perspective. *Polar Biology*, 37(7), 1041–1054.

Skarin, A., Sandström, P., & Alam, M. (2018). Out of sight of wind turbines-Reindeer response to wind farms in operation. *Ecology and Evolution*, 8(19), 9906–9919.

Skum, E. R., Turi, J. M., Moe, L., Eira, I. M. G., & Mathiesen, S. D. (2016) Reinoksens og kastratens rolle i reinflokken. In T.A. Benjaminsen, I. M. Gaup Eira, & M. N. Sara (Eds.), *Samisk reindrift. Norske myter* (pp. 129–142). Fagbokforlaget.

SMHI. (2021a). Länsvisa klimatanalyser. Antal dagar med snötäcke, minst 5 mm vatteninnehåll. https://www.smhi.se/klimat/framtidens-klimat/lansanalyser/sweden/days-snow-5-mm [accessed 2022-02-28]

SMHI. (2021b). Nollgenomgångar. https://www.smhi.se/klimat/klimatet-da-och-nu/klimatindex/nollgenomgangar-1.22895 [accessed 2022-02-28]

SMHI. (2021c). Sveriges klimat har blivit varmare och blötare. https://www.smhi.se/kunskapsbanken/klimat/sveriges-klimat/sveriges-klimat-har-blivit-varmare-och-blotare-1.21614 [accessed 2022-02-28]

Stevenson, S. K. (1990). Managing second-growth forests as caribou habitat. *Rangifer*, 10(3), 139.

Svensson, J., Andersson, J., Sandström, P., Mikusiński, G., & Jonsson, B. G. (2019). Landscape trajectory of natural boreal forest loss as an impediment to green infrastructure. *Conservation Biology*, 33(1), 152–163.

Swedish National Forestry Inventory (2021a). Table 3.1b - Productive forest area by Year (Five year average), County, Table contents and Forest type (1955-2017). Forest Statistics. [accessed 2022-02-28]

Swedish National Forest Inventory (2021b). Figure 3.17 - Growing stock for different tree species. Productive forest land (1953 - 2018). Forest Statistics. [accessed 2022-02-28]

Swedish National Forestry Inventory (2021c). 01. Notified areas of final felling by region and ownership class. Productive forest land (1995-2021). Forest Statistics. [accessed 2022-02-28]

Swedish Forestry Agency (2014). Skogsstyrelsens föreskrifter och allmänna råd till Skogsvårdslagen (SKSFS 2011:7). Skogsstyrelsen.

Wern, L. (2012). Extrem nederbörd i Sverige under 1 till 30 dygn, 1900 – 2011 (Meteorologi 143-2012). SMHI.

Other references

Total number: 16

Allard, C., & Brännström, M. (2021). Girjas reindeer herding community v. Sweden: Analysing the merits of the girjas case. *Arctic Review on Law and Politics*, 12, 56–79.

Beach, H. (1981). Reindeer-herd management in transition the case of Tuorpon Saameby in northern Sweden.

Beach, H. (1990). Comparative Systems of Reindeer Herding. In J. G. Galaty & D. L. Johnson (Eds.), *The World of Pastoralism. Herding Systems in Comparative Perspective*. (pp. 255–298). The Guilford Press.

Boda, C. S., O'Byrne, D., Harnesk, D., Faran, T., & Isgren, E. (2021). A collective alternative to the Inward Turn in environmental sustainability research. *Journal of Environmental Studies and Sciences*, 1–7.

Briske, D. D., Fuhlendorf, S. D., & Smeins, F. E. (2003). Vegetation dynamics on rangelands: a critique of the current paradigms. *Journal of Applied Ecology*, 601-614.

Ellis, J. E., & Swift, D. M. (1988). Stability of African Pastoral Ecosystems: Alternate Paradigms and Implications for Development. *Journal of Range Management*, 41(6), 450.

Fynn, R. W. S. (2012). Functional resource heterogeneity increases livestock and rangeland productivity. *Rangeland Ecology and Management*, 65(4), 319–329.

Hansen, L. I., & Olsen, B. (2014). Hunters in transition: an outline of early Sámi history. Brill.

Harnesk, D., & Isgren, E. (2021). Sustainability as a Real Utopia – heuristics for transformative sustainability research. *Environment and Planning E: Nature and Space*.

Hobbs, R. J., Hallett, L. M., Ehrlich, P. R., & Mooney, H. A. (2011). Intervention Ecology: Applying Ecological Science in the Twenty-first Century. *BioScience*, 61(6), 442–450.

Isaksen, K. R. (2018). Without foundation or neutral standpoint: using immanent critique to guide a literature review. *Journal of Critical Realism*, 17(2), 97–117.

Longo, S. B., Isgren, E., Clark, B., Jorgenson, A. K., Jerneck, A., Olsson, L., Kelly, O. M., Harnesk, D., & York, R. (2021). Sociology for sustainability science. *Discover Sustainability*, 2(1), 1–14.

Persson, S., Harnesk, D., & Islar, M. (2017). What local people? Examining the Gállok mining conflict and the rights of the Sámi population in terms of justice and power. *Geoforum*, 86, 20-29.

Sayre, N. (2017). The Politics of Scale: A History of Rangeland Science. The University of Chicago Press.

Scoones, I. (2021). Pastoralists and peasants: perspectives on agrarian change. *The Journal of Peasant Studies*, 48(1), 1-47.

Visseren-Hamakers, I. J., Razzaque, J., McElwee, P., Turnhout, E., Kelemen, E., Rusch, G. M., ... & Zaleski, D. (2021). Transformative governance of biodiversity: insights for sustainable development. *Current Opinion in Environmental Sustainability*, 53, 20-28.
